# Effect of Liuzijue Qigong on patients with chronic obstructive pulmonary disease: Protocol for a systematic review and meta-analysis

**DOI:** 10.1097/MD.0000000000012659

**Published:** 2018-10-05

**Authors:** Yu Guo, Mingmin Xu, Meiqi Ji, Jialei Zhang, Qingchuan Hu, Zeren Wei, Jian Yan, Yue Chen, Jiaxuan Lyu, Xiaoqian Shao, Ying Wang, Jiamei Guo, Yulong Wei

**Affiliations:** a School of Acupuncture-Moxibustion and Tuina, Beijing University of Chinese Medicine, Beijing; b Ovation Health Science and Technology Co. Ltd, ENN Group, Langfang; c School of Acupuncture-Moxibustion and Tuina, Chengdu University of Traditional Chinese Medicine, Chengdu; d Department of Ophthalmology, China-Japan Friendship Hospital, Beijing, China.

**Keywords:** chronic obstructive pulmonary disease, Liuzijue Qigong, protocol, systematic review

## Abstract

**Background:**

Chronic obstructive pulmonary disease (COPD) is a leading cause of morbidity and mortality worldwide with a substantial and increasing social and economic burden. Liuzijue Qigong is a kind of traditional Chinese Qigong exercises that Traditional Chinese Medicine practitioners prescribe to individuals with COPD to strengthen the internal organs’ function. Liuzijue Qigong was recommended for use in COPD rehabilitation, and some clinical trials indicate that Liuzijue Qigong would produce better functional capacity and quality of life of individuals with COPD. The objective of this study is to conduct a systematic review of the existing studies to assess effectiveness and safety of Liuzijue Qigong for the prevention or treatment of COPD in patients.

**Methods:**

We will perform the comprehensive literature search in English and Chinese electronic database. The publication period will be from inception to the search date. In addition, the clinical trial registries, dissertations, informal publication, grey literature, reference lists of studies, systematic reviews, and conference abstracts will also be collected. Two reviewers will identify relevant studies, extract data information, and then assess the methodical quality by the Cochrane risk of bias assessment tool. Only randomized controlled trials comparing Liuzijue Qigong against other intervention or nonintervention will be included. Data will be synthesized by either fixed-effect or random-effect model regarding to a heterogeneity test. The routine lung function, arterial blood gas tensions, partial pressure of carbon dioxide, functional capacity, 30 seconds sit-to-stand test, respiratory function, maximal inspiration pressure, maximal expiratory pressure, airway resistance, and specific airway conductance will be assessed as primary outcomes. The secondary outcomes involved dyspnea, and fatigue levels, respiratory muscle strength, upper and lower limb muscle strength, handgrip strength test, and health-related quality of life and safety. Meta-analysis will be performed by using Cochrane's Review Manager software (version 5.3.5).

**Results:**

This systematic review and meta-analysis will provide a high-quality synthesis and evaluate the efficacy and safety based on current relevant literature evidence of Liuzijue Qigong intervention for COPD patient.

**Conclusion:**

Our systematic review will provide evidence to determine whether Liuzijue Qigong is an effective and safe approach to prevention and treatment of COPD patients.

## 1 Introduction

Chronic obstructive pulmonary disease (COPD), as an abnormal inflammatory lung condition characterized by nonreversible persistent and progressive airflow limitation and concomitant respiratory symptoms, is a common, preventable, and treatable disease.^[1,2]^ In addition to respiratory symptom, COPD is also associated with extra-pulmonary manifestations, such as the symptoms of low weight, malnutrition, muscle weakness, pulmonary hypertension, pulmonary heart disease, pulmonary encephalopathy, anxiety, depression, induced exercise capacity impairment, decreased quality of life, and even death.^[3–5]^ COPD is a leading cause of morbidity and mortality worldwide with a substantial and increasing social and economic burden.^[1,2,6,7]^ According to a report of the World Health Organization (WHO) Global Burden of Disease Project estimated that^[1,8–10]^ the prevalence of COPD is increasing globally and it is projected to be not only the third-leading cause of death but also the seventh-leading cause of disability-adjusted life years lost worldwide by 2030, representing an important public health challenge. In the United States (US), 12 billion dollars is spent annually on hospital care for people with COPD.^[11]^ This indicates that there is an urgent need to develop effective, affordable, and alternative strategies to prevent and treat COPD.

At present, although appropriate pharmacologic therapies and surgical treatments have been proven to be effective in relieving symptoms, reducing the frequency and severity of exacerbations, and improving health status and exercise capacity of patients with COPD.^[12–15]^ However, many patients continue to suffer from dyspnea and substantial limitations in daily activities, they are often trapped in a vicious cycle of inactivity, initiated by breathlessness. Besides, the cost and adverse effects of pharmacologic therapies and surgical treatments are still never being ignored.^[12–16]^ More and more experts are beginning to realize the importance of pulmonary rehabilitation for COPD patients. Exercise training, as the cornerstone of pulmonary rehabilitation programs, have been shown to be effective in controlling symptoms, alleviating the progression, improving the exercise capacity and skeletal muscle function, and improving quality of life of individuals with COPD.^[14,15,17,18]^ Controlled breathing techniques, such as inspiratory muscle training, diaphragmatic breathing, and pursed-lip breathing, have long been incorporated as treatment components in pulmonary rehabilitation programs.^[19–24]^ However, studies have shown that respiratory training does not improve exercise tolerance or maximal oxygen uptake,^[24]^ although it improves inspiratory muscle function in COPD patients. This may be attributable to the fact that respiratory training cannot improve exertion dyspnea in COPD patients.^[25]^ What's more, some debilitated patients with COPD are unable to sustain an adequate training intensity and duration because of the rapid onset of fatigue during the initial stages of the exercise.^[26,27]^ At same time, conventional exercise training usually conducted by various facilities, including treadmill, cycle ergometer, weight training machines, and other types of equipment,^[21,28,29]^ brings a huge burden to patients, patients’ family, and social healthcare resources.^[30,31]^ Hence, convenient and cost-effective exercise training methods should be explored for COPD patients.

Recently, several investigations have reported the benefit of using complementary and alternative medicine (CAM) includes Traditional Chinese Medicine (TCM).^[32–36]^ Surveys conducted in the US and Australia have found that 60% of adults, especially older adults, employed at least 1 form of CAM for managing their chronic conditions as a complement to mainstream Western medical interventions.^[32,37]^ As we know, Qigong or chi kung (pronounced “chee-gong”) is one of the essential elements of Traditional Chinese exercises. Qigong has long been regarded as a form of mind–body intervention that simultaneously exercises the mind and the body for the treatment of various chronic diseases. Which combine the advantages of respiratory training with skeletal muscle training, can significantly improve the function of respiratory muscles and skeletal muscle, patient exercise capacity, psychological function, and quality of life.^[38–43]^ As a traditional Chinese mind–body aerobic exercise, Qigong is originally a form of ancient martial arts and is based on Taoist philosophy and TCM theories^[38,39,44]^ that has been recognized as a “medical” exercise and used to improve physical and psychological health and combat diseases in China for thousands of years.^[44,45]^ The characteristic of Qigong is self-directed and which is a combination of postures relaxation, breathing regulation, meditation, concentration, and gentle movements designed to improve holistic health and to facilitate mind–body integration.^[38–45]^ Qigong allows the exerciser to strengthen and gain control over Qi, the life-energy that flows in channels (meridians) in the body and its aims to achieve a harmonious flow of vital energy (Qi), blood, and fluid throughout the body by long-term practicing to relieve pathological stagnation and regulate the functional activities of meridians and visceral organs.^[38,39,44–46]^ With regular practice and rehearsal of the structured postures or movements, as well as concentration on mind and breath, practitioners can achieve an efficiency of “body relaxation and mind calm” and Tian Ren He Yi (the theory that mankind is an integral part of nature) so as to experience mood stabilization and improved strength and fitness.^[47–49]^ Due to its ease of learning, Qigong is appropriate for nearly anyone of any age or physical condition. What's more, it can be practiced any place and any time, without any special equipment.^[38,39,41–45,49,50]^ Therefore, Qigong is an emerging health practice that exhibits potential to be of benefit in several chronic conditions, especially for COPD.

Interestingly, Qigong is a Chinese word that other means “breathing exercise.” According to TCM theory, the “Qi” of Qigong represents the air we breathe, as well as our internal vital life energy, also known as “Yang Qi.”^[38,39,44,46]^ TCM theory states that when one's body energy is reinforced by adequate exercise, it becomes more resistant to disease.^[38,44,45]^ Qigong as a low-intensity aerobic training intervention is widely loved by the elderly, of which Liuzijue Qigong, is also called the Six Syllable Formula exercise, is considered as one of the most widely practiced forms of traditional Chinese Qigong exercises that TCM practitioners prescribe to individuals with COPD to strengthen the function of different internal organs during remission.^[46,51,52]^

Liuzijue Qigong is featured by diaphragmatic breathing and pursed lip breathing can make COPD patients breathing rate slowly and prevent the premature airway occlusion caused by rapid airflow to improve the abnormal breathing pattern of patients.^[51,52]^ In addition, Liuzijue Qigong is performed by expiration in producing 6 different sounds (“XU,” “HE,” “HU,” “SI,” “CHUI,” and “XI”) together with corresponding body movements, which not only is beneficial to the function of the auxiliary breathing muscles, but also can enhance the flexibility, coordination, and control capacity on neuromuscular of limbs to further improve exercise capacity in patients.^[46,51–53]^

Compared with conventional exercise training, Liuzijue Qigong exercise characterized by interplay between symmetrical physical postures and movements, breathing control, a meditative state of mind, and mental focus in a harmonious manner.^[46,51–53]^ Simplicity and popularity are another feature of Liuzijue Qigong, exercising do not depend on place and equipment, whatever sex or different age levels.^[51–53]^ The people can select different routine of Liuzijue Qigong according to one's requires and conditions. In addition, Liuzijue Qigong exercise can be comparatively easy to learn with less physical and cognitive demands; therefore, it is also suitable for populations with physical or cognitive impairments induced by COPD. In the preliminary searches of the electronic databases, Liuzijue Qigong was recommended for use in COPD rehabilitation, and some clinical trials were conducted to evaluate the effects of practicing Liuzijue Qigong on the physical and psychosocial functions of individuals with COPD. It was hypothesized that Liuzijue, as an alternative exercise program, would produce better functional capacity and quality of life than conventional management.^[41,51–62]^ However, while evidence arising from these individual studies have reported that Liuzijue Qigong is beneficial for patients with COPD, its definite effectiveness remains unclear due to the limitation of funding and practical clinics. To best our knowledge, there have been no relevant systematic reviews and meta-analyses on the effects of Liuzijue Qigong on prevention and treatment of COPD in patients. Moreover, despite the growing number of studies assessing the improvement of Liuzijue Qigong for COPD, the absence of critically appraised evidence continues to exist, leaving little clarity for evidence-based clinical practice. The following questions are inconclusive: how exactly is the effectiveness of standalone intervention, Liuzijue Qigong for COPD; how is additional therapeutic effect of Liuzijue Qigong when as an adjunctive treatment to conventional therapy for COPD; how long and how often is Liuzijue Qigong recommended to practice at least. Considering the substantial number of studies produced over the last decades on the health benefits of practicing Liuzijue Qigong, it was necessary and timely to perform a systematic review and meta-analysis to summarize and critically evaluate clinical trial evidence for the effectiveness of Liuzijue Qigong as complementary therapy for COPD. This English review study taking Liuzijue Qigong as an example, aim to fill the gap in the literature and provide a comprehensive synthesis of up-to-date evidence of Liuzijue Qigong in English or Chinese language studies as well as better understand current trends in the comparative effectiveness and safety of Liuzijue Qigong intervention, alone or in any combination, for patients with COPD, which may offer valuable information for the need of clinicians, patients, investigators, and future research.

## 2 Objectives

The aim of this systematic review is designed to perform a systematic assessment of the effectiveness and safety of Liuzijue Qigong for the prevention and treatment of COPD in patients.

## 3 Methods and analysis

This systematic review and meta-analysis protocol has been registered on international prospective register of systematic review (PROSPERO) as CRD42018108122 (https://www.crd.york.ac.uk/prospero/display_record.php?RecordID=108122). The procedure of this protocol will be conducted in accordance with the Cochrane Handbook for Systematic Reviews of Interventions and the Preferred Reporting Items for Systematic Reviews and Meta-Analysis Protocol (PRISMA-P) statement guidelines.^[63]^ We will describe the changes in our full review if needed.

### 3.1 Inclusion and exclusion criteria for study

#### 3.1.1 Types of studies

This review will include all parallel clinical randomized controlled trials (RCTs) that evaluated the safety of Liuzijue Qigong and its effect on patients with COPD without any language or date of dissemination or publication status restrictions. Non-RCTs, quasi-RCTs, case series, case reports, crossover studies, uncontrolled trials, and laboratory studies will not be considered.

#### 3.1.2 Types of participants

The target population is people with a confirmed clinical diagnosis of COPD (as diagnosed using any recognized diagnostic criteria, such as Global Initiative for Chronic Obstructive Lung Disease, or the British Thoracic Society, the American Thoracic Society, the European Respiratory Society or Chinese COPD guideline, or COPD was defined by the criteria of the WHO, and so on) will participant without considering any information related to their age, gender, race, education, nationality, or economic status. Depending on the number of studies retrieved, we will also consider studies in which COPD diagnosis relies on self-report.

Studies that include a mixed population where people with COPD are not the primary diagnosis will be excluded if data cannot be attained separately for people with COPD.

#### 3.1.3 Types of interventions

##### 3.1.3.1 Experimental interventions

The experimental group will receive any types of Liuzijue Qigong exercise training based on routine regimens. In addition, there is no limitation to the intervention duration and frequency.

##### 3.1.3.2 Control interventions

In the control groups, we plan to use the categories: no treatment or exercise, drug therapy or routine treatment, or conventional exercise such as jogging or walking, or other forms of Qigong such as Tai Chi Qigong, Baduanjin Qigong, and Wuqinxi Qigong. We will include studies that have compared Liuzijue Qigong plus another therapy with the same other therapy alone. At the same time, we will also exclude RCTs which have compared Liuzijue Qigong with TCM, moxibustion, acupuncture, and other TCM treatment.

The following treatment comparisons will be addressed:

1. Liuzijue Qigong versus no treatment or exercise
2. Liuzijue Qigong versus drug therapy or routine treatment
3. Liuzijue Qigong versus other conventional exercise
4. Liuzijue Qigong versus other forms of Qigong
5. Liuzijue Qigong plus another therapy versus the same other therapy alone

#### 3.1.4 Types of outcome measures

##### 3.1.4.1 Primary outcomes

The primary outcome measurement will be an improvement of routine lung function (forced expiratory volume in 1 second and forced vital capacity), arterial blood gas tensions (arterial oxygen pressure, partial pressure of carbon dioxide), functional capacity (6-minute walk test), 30 seconds sit-to-stand test, respiratory function, maximal inspiration pressure, maximal expiratory pressure, airway resistance, and specific airway conductance.

##### 3.1.4.2 Secondary outcomes

The secondary outcomes involved dyspnea, and fatigue levels, respiratory muscle strength, upper and lower limb muscle strength, handgrip strength test, health-related quality of life (health-related quality of life measurements using validated tools, such as general and mental health subscales of the Medical Outcomes Study 36-Item Short Form Health Survey, St. George's Respiratory Questionnaire, Chinese Chronic Respiratory Questionnaire, and so on) and safety that measured by incidence and severity of side effects associated with use of Liuzijue Qigong for prevention or treatment in patients with COPD.

### 3.2 Search strategy for the identification of studies

#### 3.2.1 Electronic searches

Based on our awareness of numbers of existing reviews and meta-analyses that can be utilized, we will perform the comprehensive literature search in both English and Chinese electronic database involving PubMed Database, Embase Database, Cochrane Library, Web of Science database, Medline, Chinese BioMedical Literature Database, China National Knowledge Infrastructure, China Science and Technology Journal database, and Wanfang Data Chinese database. The publication period will be from inception to the search date. According to the Cochrane Handbook Guidelines, all the reviewers will discuss the search terms and search strategies. The following search terms will be adopted: “Chronic Obstructive Pulmonary Disease” OR “Pulmonary Disease” OR “Chronic Obstructive” OR “Pulmonary Emphysema” OR “Emphysema” OR “COPD” OR “COAD” OR “COBD” OR “AECB” OR “Chronic Obstructive Pulmonary” OR “Chronic Obstructive Airway” OR “Chronic Obstructive Lung” OR “Chronic Obstructive Bronchopulmonary” OR “Chronic Obstructive Respiratory” OR “Chronic Airflow Obstruction” OR “Chronic Airflow Obstructive” OR “Chronic Bronchitis” OR “Pulmonary Emphysema” OR “Lung Emphysema” OR “Chronic Airflow Limitation” OR “Chronic Obstructive Airway Disease” OR “Chronic Obstructive Lung Disease” OR “Lung Diseases” OR “Chronic Airway Obstruction” AND “Qigong” OR “Traditional Chinese Exercise” OR “Mind–body Exercise” OR “Respiratory Training” OR “Respiratory Exercise” OR “Breath Training” OR “Breath Exercise” OR “Breathing Technique” OR “Liuzijue” OR “Liuzijue exercise” OR “Liuzijue Qigong” OR“ Six Syllable Formula.” The equivalent search terms will be translated into Chinese while searching in the Chinese databases. The list of proposed search terms will be developed with the assistance of a health science librarian and reviewed by a number of experts in the fields of physiotherapy and lung diseases and specific Qigong specialists, any necessary adjustments made prior to running the search. Example of detailed search strategies for PubMed is shown in Table 1.

**Table 1:**
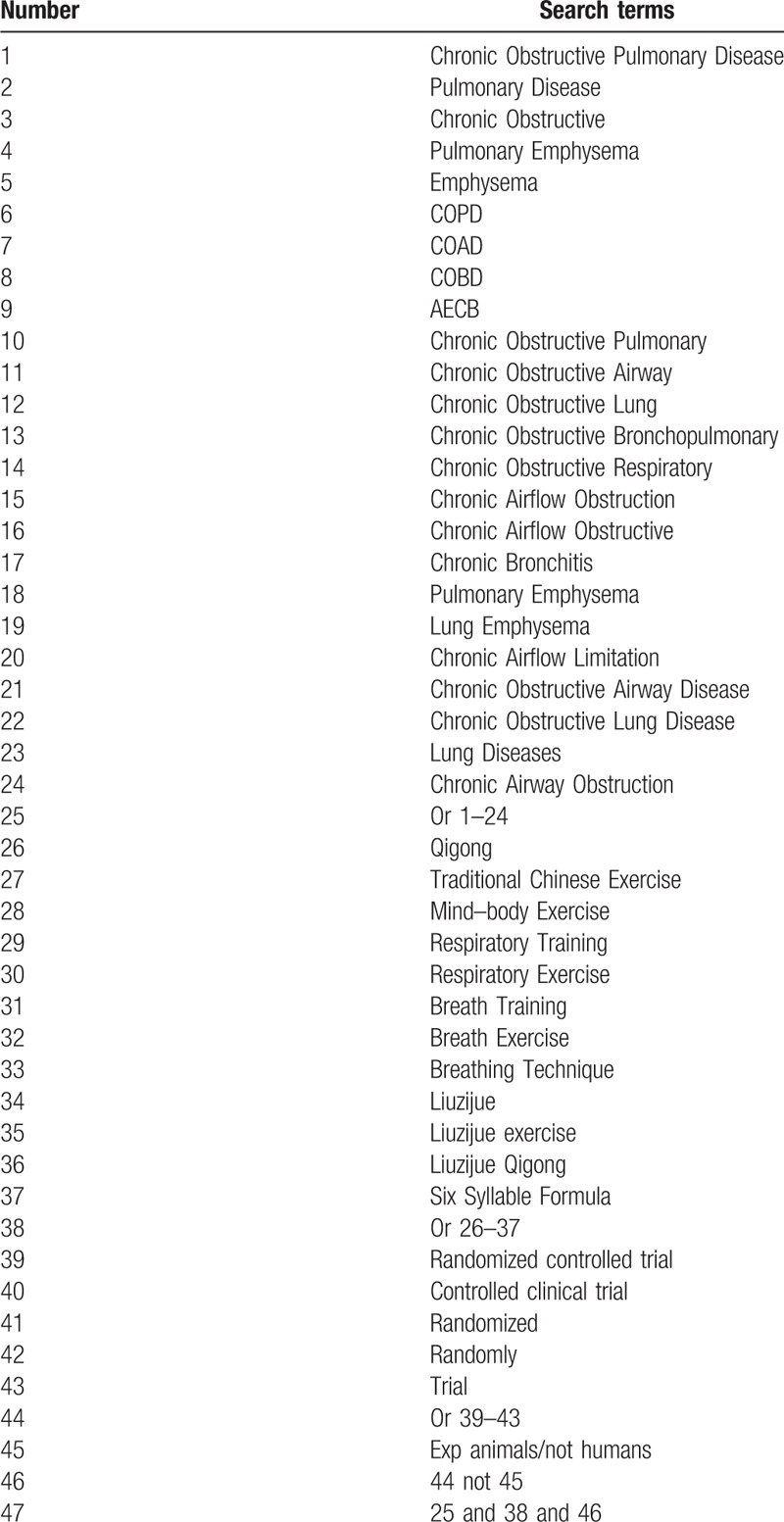
Search strategy for the PubMed database.

#### 3.2.2 Searching other resources

In addition, the clinical trial registries, dissertations, informal publication, and grey literature will be searched from inception to the search date. For additional trials, we will search the reference lists of studies, systematic reviews, and conference abstracts related to Liuzijue Qigong and COPD. Ongoing trials for the new reviews, which relevant to this term, will be retrieved from the WHO International Clinical Trials Registry Platform, Current Controlled Trials, US National Institutes of Health Ongoing Trials Register, Australian New Zealand Clinical Trials Registry, and the Chinese Clinical Trial Registry. We will also search in OpenGrey.eu. website for potential gray literature. We will include relevant conference abstracts if all information can be retrieved; if information is missing we will contact the study leaders for additional information. If sufficient information is provided, conference abstracts will be included in the analyses; if not, they will be excluded. Also, the study leaders of identified unpublished and studies in progress will be contacted to establish whether published literature was missed.

### 3.3 Data collection and analysis

#### 3.3.1 Selection of studies

**Figure 1:**
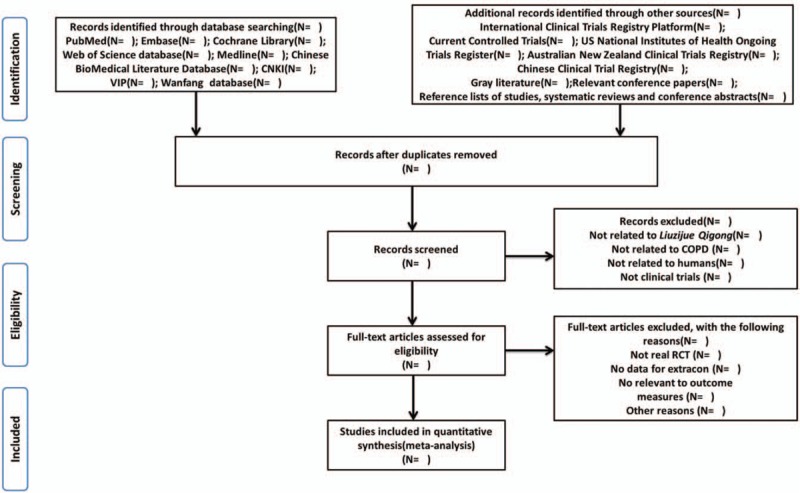
Preferred Reporting Items for Systematic Reviews and Meta-Analyses (PRISMA) flow diagram. The PRISMA statement is used worldwide to improve the reporting of systematic reviews and meta-analyses.

Before selection of studies, all reviewers must get trained in order to understand the purpose and process of the review. We will exclude articles for which no data on COPD outcomes is presented, relevant information is unavailable, or results are duplicated. Two reviewers (Yu Guo and Mingmin Xu) will identify all potential relevant studies and sequentially screen their titles, abstracts, and keywords for eligibility independently after removing duplications. The further examining will be performed to select eligible articles by reviewing the full-text and considering for analysis. All reviewers will screen the name of the author, institution, and journal of publication. And a PRISMA flow chart will be produced to show the number of articles identified, screened, included, and excluded, reasons for exclusion and to ascertain eligible studies. Any discrepancies will be resolved through discussion to get a consensus, for example, through plenary meetings or discussions by email or will be judged by a third reviewer (Meiqi Ji). The study selection procedure will be described in a PRISMA flow chart (Fig. 1).

#### 3.3.2 Data extraction and management

According to the inclusion, a standard data collection form will be made before data extraction. For studies fulfilling the inclusion criteria, 2 reviewers (Yu Guo and Mingmin Xu) will then independently extract data from the selected studies about the general information, methodological description, study population characteristics, intervention characteristics, intervention characteristics, outcome measures, and notes and fill in a self-developed data extraction form. Any disagreements will be resolved through consultation and if necessary, the discrepancies and uncertainties will be resolved to get a consensus by consulting a senior reviewer (Meiqi Ji) through discussion. We will contact the corresponding authors for more information if the details of the trials were not completed. All data will be cross-checked by Yu Guo and Mingmin Xu and transferred into Review Manager Software (RevMan). To maintain consistency, results reflect outcome measures immediately after program completion. The following data will be extracted:

1. General information: Research identification, publication year, the title of the study, name of first author, correspondent author, contact information, the name of the journal, and country of origin.
2. Study methods: Aims and objectives, study design, sample size, randomization method, allocation concealment, details of blinding methods, incomplete report or selecting report, and other sources of bias.
3. Participants characteristics: Recruitment and sampling methods, inclusion and exclusion criteria, mean age, gender, ethnicity, status of COPD, duration of disorder, severity of disorder, research setting, current treatment, the baseline of COPD, diagnostic criteria used of COPD if reported.
4. Intervention characteristics: Type of intervention, program period, frequency, session durations, who supervised the program (if applicable), chemotherapy drugs, routes of administration other treatment, treatment details, treatment duration, and frequency.
5. Outcomes data: Primary, secondary, and safety outcomes as described above, length of follow-up, timing of follow-up measures, and the key findings of the study, including both within-group and between-group differences.
6. Notes: Financial support, conflicts interest, ethical approval, and important citations.

#### 3.3.3 Assessment of ROB in included studies

The methodical quality in included studies will be evaluated independently by 2 reviewers (Yu Guo and Mingmin Xu) according to the risk of bias (ROB) assessment tool provided by the Cochrane Handbook for Systematic Reviews of Interventions.^[64]^ The following categories of risk and bias will be assessed: selection bias (random sequence generation and allocation concealment), detection bias (blinding of outcome assessors and statisticians), attrition bias (incomplete outcome data), reporting bias (selective outcome reporting), and other bias. Bias among participants and investigators will not be considered because the Liuzijue Qigong make blinding impossible. We will give each item 3 potential bias judgments: low bias, unclear (unclear or unknown ROB), or high bias. A clinical trial meeting all criteria will be judged as having a low ROB, a trial meeting none of the criteria will be judged as having an ROB, and a trial with insufficient information to judge will be classified as unclear ROB.

Any discrepancies will be resolved by a discussion between the 2 reviewers or consulting a third reviewer (Meiqi Ji) where necessary. On completion of this process, the remaining studies will be assessed for ROB by 1 reviewer. Overall, the quality assessment will consider the following aspects:

(1) Adequate sequence generation: Whether the allocation sequence was generated appropriately.
(2) Concealment of allocation: Whether study participants and research staff were unaware of the intervention given at the enrolment stage.
(3) Blinding of outcomes: Whether the personnel assessing outcomes and analyzing the data were blinded to the intervention allocated.
(4) Adequately addressed incomplete outcome data: Were incomplete outcome data adequately reported in the published study?
(5) Free from selective reporting: Whether the outcomes were reported selectively.
(6) Other sources of bias: Whether the study was apparently free of any other high ROB (such as funding and potential for conflict of interest).

#### 3.3.4 Measures of treatment effect

Effect size is known to be an appropriate index to combine various intervention effects retrieved from different studies. The conventional pair-wise meta-analysis will be performed using random-effect model by RevMan V.5.3.5. For continuous data, we will use mean difference (MD) or standardized MD with 95% confidence interval (CI). For dichotomous outcomes, we will use the relative risk (RR) and 95% CI records.

#### 3.3.5 Unit of analysis issue

We will assess the first experimental period data in cross-over trials to prevent carry-over effects. With multiple intervention groups, we will combine all relevant experimental and control intervention groups into a single, respectively, to avoid a unit of analysis issue.

#### 3.3.6 Dealing with missing data or unclear measurement scales

If data information is missing in the included studies, 2 reviewers (Yu Guo and Mingmin Xu) will contact leaders of articles by email or telephone to request missing data or additional information. If sufficient information cannot be obtained in this way, we will analyze the available data and describe it in the discussion. We will discuss the impact of missing data if necessary and take into account the potential impact of insufficient data on the review results (Section 4).

#### 3.3.7 Assessment of heterogeneity

If we find our included studies (or groups of studies) are sufficiently homogenous in terms of design, study population, and outcomes, we will use complete case data as analysis data. Heterogeneity will be assessed by a standard chi-squared test with a significance level of *P* < .1 and I^2^ test according to the guidelines in Cochrane Handbook for Systematic Reviews. When the I^2^ value is <50%, the study will be considered to have no statistical heterogeneity, and the fixed-effect model will be selected. While I^2^ ≥ 50%, the study will be considered to have substantial heterogeneity, and we will select a random-effect model.

#### 3.3.8 Data synthesis and analysis

We will synthesize and analyze the date using RevMan V.5.3.5 provided by Cochrane Collaboration. Heterogeneity across studies was tested by the Ι^2^ statistic. In the Cochrane Handbook, when I^2^ < 50%, a fixed-effect model will be used to calculate the RR and MD, when I^2^ ≥ 50% was regarded as may material heterogeneity and we will use a random-effect model to synthesize the data. Subgroup analysis will be performed and the potential reasons will be analyzed to explore the causes of heterogeneity. If meta-analysis is not appropriate, we may use narrative synthesis.

#### 3.3.9 Assessment of reporting bias

When 10 or more studies are included in the meta-analysis, a funnel plot and statistic test will be developed to evaluate reporting bias of the included studies.

#### 3.3.10 Subgroup analysis

If the substantial heterogeneity or inconsistency among the studies was detected and to explore whether the effects are different in different subgroups, we will perform subgroup analysis for different COPD measurements and intervention forms. We will take study quality, durations, frequencies, types or forms of Liuzijue Qigong, the degree of COPD severity, the age and sex of patients, geographical area, and other different control interventions into consideration. If sufficient studies are identified, we will also calculate the incidence rates of different types of adverse events.

#### 3.3.11 Sensitivity analysis

We will perform sensitivity analysis for primary outcomes to explore the robustness of the review conclusions if feasible, and we will still evaluate the impact of methodological quality, sample size, and missing data. Different levels of the methodological quality of studies will influence the overall effects. Sensitivity analysis will be conducted by removing trails that report the nonrandom sequence generation.

#### 3.3.12 Grading the quality of evidence

The Grading of Recommendations Assessment, Development, and Evaluation guidelines^[65]^ will be used to evaluate the quality of evidence and confidence for primary outcomes in including studies. Reviewers will take into account limitations of the study, inconsistencies of effect results, indirect evidence, inaccuracies, and publication bias. Additional domains may be considered if deemed appropriate.^[30]^ The quality of evidence will be classified into “very low,” “low,” “moderate,” or “high” judgment. Strength of evidence will be judged as “high” (further research is unlikely to change our conclusion), “moderate” (further research is likely to alter our conclusion), or “low” and “very low” (further studies are required to answer the research question with a high degree of confidence/increase confidence).

#### 3.3.13 Ethics and dissemination

Our findings will summarize the evidence on the effectiveness and safety of Liuzijue Qigong for physical and psychological well-being, quality of life in patients with COPD. Formal ethical approval is not required as this study is a systematic review bases on the published evidence and all data used in this study will be anonymous with no concerns regarding privacy. This is the protocol for a systematic review and meta-analysis; there is no patient and public involvement. It is our intention to submit the results of our review for peer-reviewed publication and to present our findings at national and international meetings and conferences.

## 4 Discussion

Over the past decade, COPD has become a major public health problem, with continuously increasing prevalence throughout the world.^[1,6,7]^ COPD is a common and incurable respiratory disease affecting the airways of the lungs resulting in loss of lung function that impacts the health and quality of life of patients worldwide.^[8–10]^ It is characterized by predominantly fixed airway obstruction through a variety of processes. The pathogenesis involves many components, including the hypersecretion of mucus, oxidative stress, and inflammation in the airway and lung.^[1,2]^ Patients with COPD frequently complain of dyspnea and exercise limitation and become trapped in a vicious cycle of inactivity, initiated by breathlessness.^[1,2]^ Although formerly considered a disease that mainly results in pulmonary impairment, it is now recognized that people living with COPD have many systemic manifestations including peripheral muscle dysfunction, right-sided heart failure, malnutrition and depression, and other organs’ disorder.^[1–5]^ The associated morbidity presents both social and economic burden because of the high levels of care required for people with COPD and individuals often do not seek medical attention until the disease is moderate or severe. So it is essential to make an appropriate strategy or method to significantly prevent and treat COPD.

Currently, treatment is mainly symptomatic and aims to slow down disease progression.^[12–16]^ Exercise training, the important part of pulmonary rehabilitation, has been shown to improve dyspnea and health status and decrease healthcare use. Undoubtedly, exercise training should be one of the vital approaches in the treatment of COPD.^[14,15,17,18,24–27]^ However, it is generally known that conventional exercise training requires equipment, an exercise location, and medical personnel to supervise its safety, which poses a heavy burden on patients’ families and health resources.^[21,28–31]^ Compared with conventional exercise modalities, Qigong is simple, soft, and relax, and the playing space and exercise equipment are not restrictive,^[38,39,41–45,49,50]^ besides, Qigong emphasizes the importance of the inner body while physical exercise focuses on the outer body.^[66]^ Qigong is a traditional form of mind–body exercise, which is an important branch of TCM and dates back thousands of years.^[38–40]^ Qi denotes vital energy that can sustain human well-being and assist in healing, and Gong means practice or skill.^[38,67,68]^ In the current definitions of Qigong^[66]^: “Qigong is the skill of body–mind exercise that integrates the 3 adjustments of body, breath, mind into ‘one’.” According to the philosophy of TCM, Qigong training is designed to strengthen and circulate the Qi (vital energy) of the overall body along the energy channels (meridians)^[38,39,44–46]^ based on integrating the 3 adjustments of body, breath, and mind into “one.”^[38,66]^ Thus, practicing Qigong simultaneously trains the body, breath, mind, and Qi (vital energy) for the benefits of physical, psychological, and spiritual health. Liuzijue Qigong, also call Six Syllable Formula, is an ancient health practice and breathing exercise Qigong (Tu-Na) in China passed down from ancient times, reinforces that the Qi plays an important role in health exercise.^[46,51,52,66]^ Because the essence of the practice is vocalizing 6 different sounds of “XU,” “HE,” “HU,” “SI,” “CHUI,” and “XI,” respectively, while exhaling the breath, it is referred to as the “Six Syllable Formula Health Practice.”^[46,51–53,55–57]^ The earliest record of this technique is found in “The Book of Documents of Shang shu.” Liuzijue Qigong is based on TCM of Yin and Yang, the 5 phases, the unity of man and nature, and engendering and restraining. This correlates the sequence of the 4 seasons with the attributes of the 5 viscera (liver, heart, spleen, lung, and kidney). It uses the shape of the mouth in the pronunciation of the 5 musical tones (Jiao, Zhi, Gong, Shang, and Yu) to correlate with the movements of the breath.^[46,66]^ The Liuzijue Qigong values the regulation of the breathing and aspiration, requiring an enhancement to the breath depth and cycle, and trying to reach a “fine, deep, long, and balance” target as far as possible and its diaphragmatic breathing may produce increased asynchronous and paradoxical breathing movements, and its prolonged expiration and slowing of the breathing rate is widely used and produces a satisfactory effect.^[46,51–53,55–57]^ The exercises feature slow and gentle movement of the mouth and tongue, which is believed to regulate the rise and fall of Qi (vital energy) through the meridian system in the body and to help nourish the corresponding organs as a low-intensity aerobic exercise, it exerted beneficial effects by reducing breathing frequency, modulating airway reactivity, increasing respiratory sensation through conditioning of the breathing pattern, reducing oxygen consumption, decreasing responses to hypoxic and hypercapnic conditions with better blood oxygenation without increasing minute ventilation, improving respiratory muscle strength and endurance at least for a short-term, and decreasing the resting heart rate and sympathetic reactivity.^[46,51–62,66]^ Therefore, Liuzijue Qigong can synchronize breathing and speech and increase respiratory muscle strength and coordination that are suitable for physically weak and elderly patients with COPD. Recently, Liuzijue Qigong was recommended for use in COPD rehabilitation, and some clinical trials were conducted to evaluate the effects of Liuzijue Qigong on patients with COPD. The present study showed that the program of Liuzijue Qigong results in improvements in pulmonary function, immunologic function, respiratory muscle strength, peripheral skeletal muscle function, exercise capacity, mental health, and quality of life in patients with COPD, especially the older adults.^[51–62]^ What's more, Liuzijue Qigong regular physical activities, in addition to having less chance of inducing muscle damage, may have a modulation effect on low-level inflammation, which was demonstrated in individuals with COPD. It has been proposed that the therapeutic effect is mediated through enhancing better circulation, which is essential for the regulation of the inflammatory and immune responses for the enforcement of the body's natural self-healing ability.^[51–62]^ In addition, Liuzijue Qigong style appeared to be safe during training and patients enjoyed the training. Furthermore, selecting Liuzijue Qigong as a health promotion program is advantageous because it can be practiced anywhere (indoors and outdoors) and requires no special equipment. Once people learn the skill, they can practice Liuzijue Qigong by themselves; it could be a valuable and cost-effective exercise regimen to enhance the well-being of people with COPD.^[51–62]^ Nevertheless, to date, there has been no comprehensive evaluation of the clinical evidence concerning the effectiveness of Liuzijue Qigong on patients with COPD based on evidence-based medicine. We aim to synthesize the relevant literature and conduct this systematic review and meta-analysis to evaluate the efficacy and safety of Liuzijue Qigong intervention for COPD patients. We expect to find that Liuzijue Qigong have a positive effect on prevention and treatment of COPD in individuals. The results of this review may help to establish a better approach to prevention and treatment of COPD in patients and to provide reliable evidence for helping patients and clinicians to make right choices when applying Liuzijue Qigong.

Despite our careful methodological considerations, it should be noted that there might be several limitations have to be mentioned regarding our systematic review and meta-analysis. First, the use of language including English and Chinese and exclusion of papers written in languages not known by the research which may induce the bias of the study. Second, diverse style of Liuzijue Qigong, time points when interventions was initiated, intensity, duration, frequency, the age and sex of patients, degree of COPD severity, and study quality may cause high statistical heterogeneity. Third, it is impossible to conduct single- or double-blind experiment measures during Liuzijue Qigong training.

## Acknowledgments

The authors would like to deeply acknowledge Professor Tianjin Liu from BUCM, Professor Qing Tang and Weibo Zhang from Ovation Health Science and Technology Co. Ltd, ENN Group for providing valuable suggestions to conduct this study.

## Author contributions

Yu Guo and Mingmin Xu are the guarantor of the article. Yu Guo and Mingmin Xu, and Yulong Wei contributed to conceive the idea of research, developed the search strategy, and drafted of the manuscript. Meiqi Ji, Jialei Zhang, and Qingchuan Hu critically revised the manuscript and provided valuable advice on the process of the protocol. Yu Guo is in charge of coordination and direct implementation. Yulong Wei is responsible for monitored the process of the study. Yu Guo and Mingmin Xu will screen the titles, abstracts, keywords of all retrieved records and extract data independently. Yue Chen and Jian Yan will assess the risk of bias independently. Zeren Wei and Jiaxuan Lyu will deal with the missing data. Ying Wang and Jiamei Guo will conduct statistical analysis Xiaoqian Shao and Meiqi Ji will arbitrate any disagreements in the review. All review authors approved the publication of the protocol. All authors participated in the protocol design, commented on drafts of this paper, and read and approved the publication of the final manuscript.

**Conceptualization:** Yu Guo, Mingmin Xu, Yulong Wei.

**Data curation:** Jialei Zhang, Xiaoqian Shao.

**Formal analysis:** Ying Wang, Jiamei Guo.

**Investigation:** Yu Guo, Mingmin Xu, Meiqi Ji, Zeren Wei.

**Methodology:** Yu Guo, Mingmin Xu, Meiqi Ji.

**Project administration:** Yu Guo, Mingmin Xu, Jialei Zhang, Yulong Wei.

**Supervision:** Yu Guo, Mingmin Xu, Yulong Wei.

**Validation:** Yu Guo, Mingmin Xu, Qingchuan Hu, Jiaxuan Lyu.

**Visualization:** Yu Guo, Mingmin Xu, Meiqi Ji, Jialei Zhang, Qingchuan Hu, Zeren Wei, Jian Yan, Yue Chen, Jiaxuan Lyu, Xiaoqian Shao, Ying Wang, Jiamei Guo, Yulong Wei.

**Writing – original draft:** Yu Guo, Mingmin Xu.

**Writing – review & editing:** Meiqi Ji, Qingchuan Hu, Jiaxuan Lyu.
